# When the Triplet State Doesn’t Matter: Insights into Its Impact on *V*_OC_

**DOI:** 10.1021/acsenergylett.5c00384

**Published:** 2025-04-23

**Authors:** Mohammad Saeed Shadabroo, Nurlan Tokmoldin, Atul Shukla, Acacia Patterson, Tanner M. Melody, Obaid Alqahtani, Brian A. Collins, Dieter Neher, Safa Shoaee

**Affiliations:** † Institute of Physics and Astronomy, University of Potsdam, Karl-Liebknecht-Str. 24-25, 14476 Potsdam-Golm, Germany; ‡ Heterostructure semiconductor physics, Paul Drude Institute for Solid State Electronics, Hausvogteiplatz 5-7, 10117 Berlin, Germany; § Department of Physics and Astronomy, Washington State University, 100 Dairy Road, Pullman, Washington 99164, United States; ∥ Department of Physics, Prince Sattam bin Abdulaziz University, Alkharj 11942, KSA

## Abstract

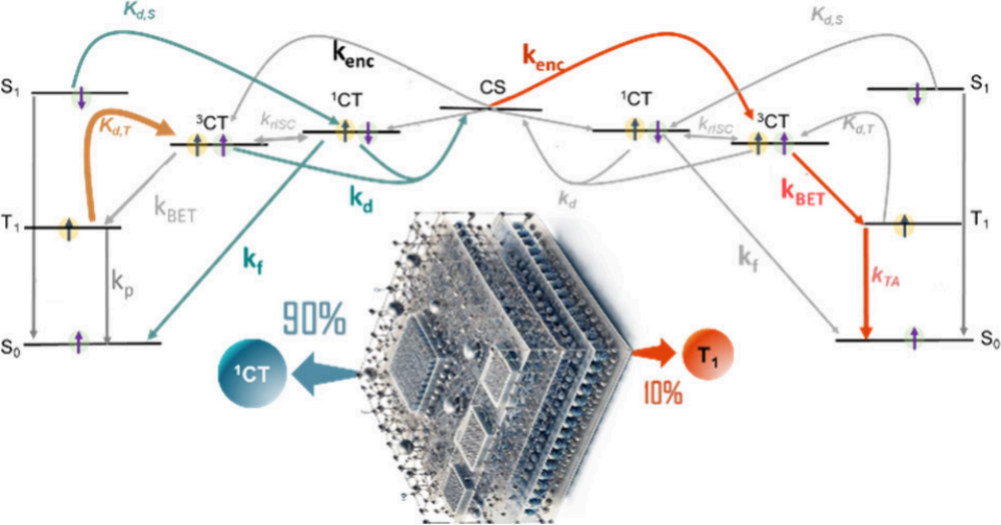

Organic solar cell efficiency, exceeding 20%, is limited by recombination losses from singlet and triplet charge-transfer (CT) states and local triplet excitons, impacting open-circuit voltage (*V*_*OC*_). Using PM6:o-IDTBR, a very low nonradiative voltage loss, Δ*V*_*nr*_ = 160 mV, is achieved, despite the presence of triplet excitons (6 × 10^15^ cm^–3^). In employing the present system as a model exemplar, we elucidate the circumstance wherein, if the triplet lifetime surpasses the lifetime of the CT decay, the dissociation of triplet excitons to the CT state emerges as a feasible process. This, in turn, serves to reduce the manifestation of an additional loss channel from the T1 state and minimizes losses from T1. In PM6:o-IDTBR, the long triplet lifetime (10 μs) enables dissociation of the triplet state and limits the triplet-mediated recombination to ∼10%.

The past five years have seen a rapid improvement in organic solar cells (OSCs), with record device power conversion efficiencies (PCE) in single junctions reaching over 20%.^1−3^ Some of this success can be attributed to the decrease in the open circuit voltage (*V*_*OC*_) losses. In fact, numerous endeavors have been made to predict design rules and strategies aimed at mitigating *V*_*OC*_ losses.^4^ These strategies include reduction of interfacial area and energy offset between the donor and the acceptor, hybridization, exciton lifetime, and photoluminescence quantum yield (PLQY) of pure nonfullerene acceptors (NFA).^5^ Considerable efforts have been invested in combining materials to meet these requirements, leading to notable gains. However, despite these advancements, significant losses still remain, suggesting that not all contributing factors have been fully considered. One key area that has largely been overlooked is the role of triplet excitons. While triplet–triplet annihilation might aid in reducing losses,^6^ triplets are otherwise perceived as an adverse state.^7,8^ Recent reports indicate that triplet states are prominently observed even in highly efficient OSC blends.^10^ However, the effect of triplets on charge carrier density and *V*_*OC*_ remains almost absent from the current body of literature. In fact, triplet behavior in OSCs stands as one of the most understudied aspects, presenting a critical void that demands urgent attention and investigation.

In organic semiconductors, optical excitation predominantly gives rise to singlet excitons (S1), characterized by a spin multiplicity of zero. Nonetheless, there exist lower energy states known as triplet excitons (T1), distinguished by a spin multiplicity of 1. The formation of triplet excitons can occur through various pathways, including direct intersystem crossing from the local S1 state, intersystem crossing between singlet and triplet charge transfer (CT) states, and charge recombination.^9^ The latter has been reported to be responsible for 90% of the nonradiative recombination of photogenerated charges in state-of-the-art PM6:Y6 and other NFA systems.^10^ However, fullerene blends have also been reported to suffer from polaron-mediated triplet formation.^11−13^

Multiple pathways exist for the emergence of a triplet exciton from the initially excited S1 state. While direct formation of the triplet exciton through intersystem crossing (ISC) from the S1 state is conceivable in both neat and blended systems, its efficiency is relatively low. In addition to low ISC efficiency, notably, in blends with a high energy offset, where the CT states possess lower energy levels than the dark triplet state, intersystem crossing from S1 to T1 is surpassed by the more efficient charge transfer process.^14^ In blended systems, the formation of triplets can occur from the CT state, either geminately or nongeminately. In the former scenario, before the ^1^CT dissociates into free charges or decays to the ground state, intersystem crossing takes place, forming a triplet CT (^3^CT) state.^14^ Subsequently, through back electron transfer (BET), the triplet exciton is generated. This results in relatively low internal quantum efficiencies. On the other hand, in the nongeminate mechanism, the recombination of initially separated charges — even with internal quantum efficiency, IQE of 100% — leads to the formation of CT states with singlet and triplet character, with a ratio of 1:3 following spin statistics.^9,13^ Singlet CT states have been reported to decay relatively fast (typically, *k*_*f*_ of 10^9^–10^10^ s^–1^). Conversely, while the decay of the ^3^CT states to the ground state is slow, due to spin-forbidden processes, when T1 is below ^3^CT, the back electron transfer rate from ^3^CT to T1 is believed to be fast.^6^ This rapid rate leads to the inevitable formation of triplets, introducing a significant loss channel. Indeed, in many BHJ systems, triplet exciton formation from ^3^CT has been identified as a major energy loss channel.^7,10,15,16^

It is generally established that the T1 state is often one of the lowest energy states of the system. Consequently, both its formation and subsequent relaxation to the ground state exert a dual impact on fill factor (FF) and voltage loss simultaneously through nonradiative recombination (as ideally the solar cell should possess only radiative recombination).^8^ The precise kinetic dynamics governing efficient nongeminate triplet exciton formation remain elusive, yet it is anticipated that the energy difference between the ^3^CT and T1 states as well as transition rates influenced by morphological factors such as delocalization and molecular packing significantly impact the process. Indeed. in numerous blend systems — such as PCPDTBT:PCBM and RR-P3HT:PCBM, BTR:PCBM — it has been observed that thermal or solvent annealing or use of additives has resulted in suppression of triplet formation.^18,19^ This suppression is likely attributable to increased crystallinity resulting in larger domains and enhanced hole delocalization.^20^ Conversely, in the DRCN5T:PC71BM blend, thermal annealing has been reported to lead to an increase in the yield of triplet excitons yet with more efficient device performance.^21^ To understand the significance of triplets in device performance, in this work we consider *V*_*OC*_. Abiding by the energy gap law, a theoretical framework elucidating transition rates in relation to energy differences between states, it is anticipated that the transition rate between ^3^CT–T1–S0 surpasses the rate of transition between ^1^CT–S0. This expectation aligns with findings corroborated by Gillett et al.^10^ However, one then encounters a perplexing contradiction. The nonradiative *V*_*OC*_ loss has been reported to be dependent on the ^1^CT–S0 gap^22^ rather than the T1–S0 gap,^23^ which goes against the expectations set by the energy gap law. This discrepancy challenges our understanding, prompting us to question the true underlying mechanisms at play. Within this study, we meticulously investigate the impact of triplets concerning *V*_*OC*_ losses in PM6:o-IDTBR devices. We determine the steady state triplet exciton population in PM6:o-IDTBR blend to be 6 × 10^15^ cm^–3^ (under continuous AM1.5G illumination) using photoinduced absorption (PIA) measurements. While PIA reveals different ratios of triplets to free charges in annealed and as-prepared devices, the carrier density and the *V*_*OC*_, however, yield the same value. Of particular interest, PIA indicates a long triplet lifetime of 10 μs for o-IDTBR. Tentative comparison of CT energy levels against temperature-dependent *J*–*V* data suggest the voltage at 0 K is primarily limited by the o-IDTBR CT state and not the triplets. Yet of particular importance is the very low nonradiative voltage loss (Δ*V*_*nr*_) reported for this blend system and the lower T1 energy level, prompting a captivating query: under what circumstances do triplet excitons avoid decay to the ground state, thus mitigating their participation in nonradiative voltage loss? Comparing several systems, we deduce PM6:o-IDTBR and PTQ10:o-IDTBR are examples of low nonradiative voltage loss systems that will provide insight into the role of triplet exciton formation on *V*_*OC*_.

Figure 1a presents the chemical structures, and panel b shows a comparative analysis of the device performance of the as-prepared and the annealed PM6:o-IDTBR (1:1.2 blend ratio) devices. Thermal annealing is demonstrated to improve the current density (*J*_*SC*_) and the fill factor with no discernible impact on the *V*_*OC*_ (see Supplementary Note 2 Table S1). To elucidate the prevailing mechanism in both the as-prepared and annealed blends, steady state absorption and photoluminescence (PL) were performed at ambient temperatures. The ground state absorption spectra of the neat o-IDTBR and blend films fabricated for the study are depicted in Figure 1c. The neat as-prepared o-IDTBR film shows a peak at 700 nm. Upon blending with PM6, a blue shift is observed, shifting the peak maximum to 650 nm. This blue-shift indicates that the introduction of PM6 causes a distortion in the crystal lattice of o-IDTBR domains, consistent with morphology micrographs (Figure 1 and Figure S1) showing o-IDTBR’s crystallinity is significantly reduced in the blend relative to pure film. This result is similar to that reported in other materials.^24^ In numerous systems, such P3HT:PCBM, thermal annealing has been documented that reinstates the crystallinity of the blend film and enhances device efficiency. In neat film, annealing induces a substantial shift in absorption (red-shifted and peak centered at 720 nm), a phenomenon also observed in other NFAs. Remarkably, this shift remains absent in the blended annealed film, exhibiting an intriguing similarity in absorption to the as-prepared blend, without any discernible shift in the peak.

**Figure 1:**
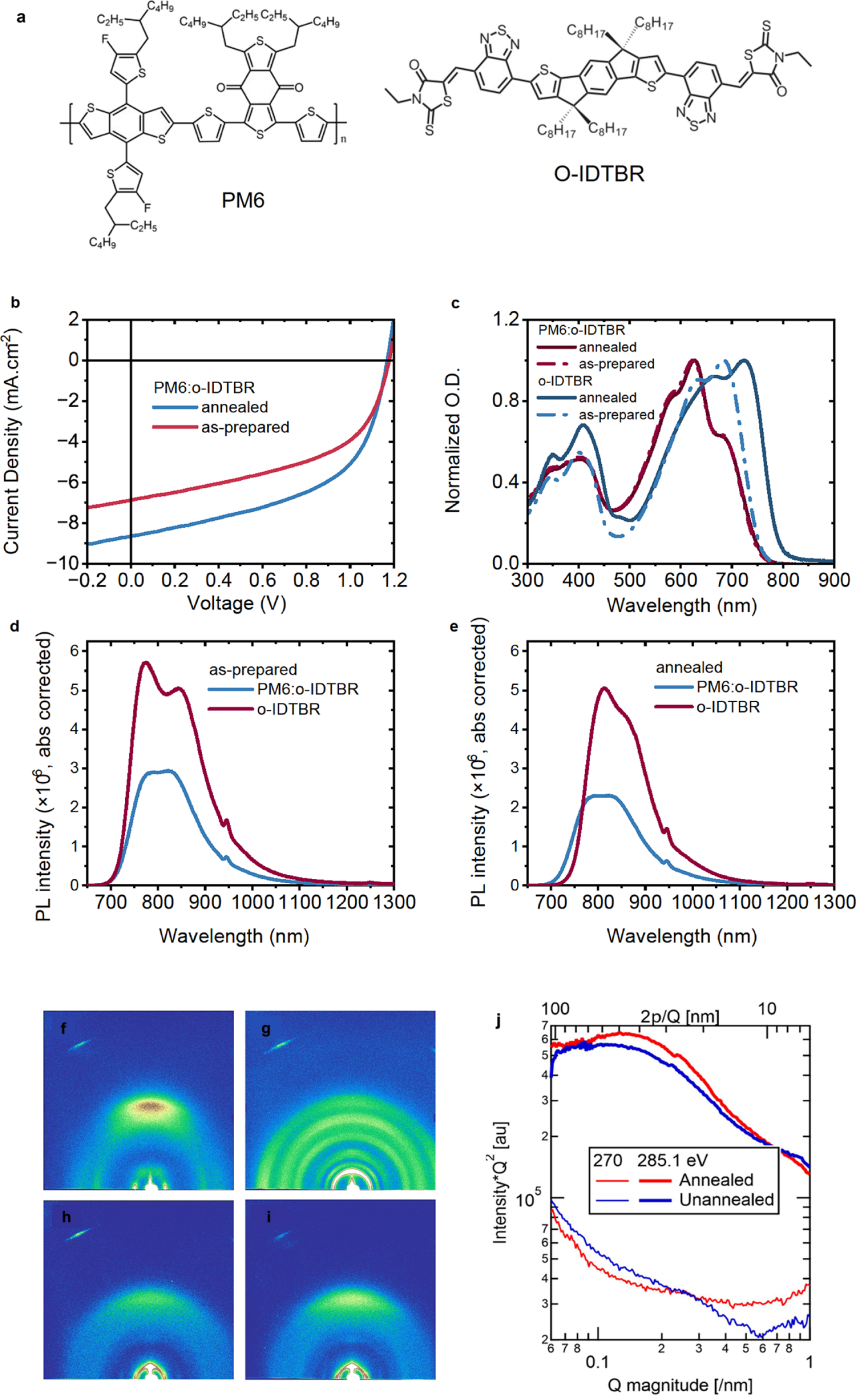
(a) Chemical structures of PM6 and o-IDTBR; (b) current density vs voltage (*J*–*V*) curves of 100 nm thick junctions of as-prepared and annealed PM6:o-IDTBR (1:1.2 blend ratio, see the Supporting Information for full device details); (c) UV–vis absorption spectra of the studied films. Photoluminescence measurements of (d) as-prepared neat o-IDTBR vs as-prepared PM6:o-IDTBR and (e) annealed neat o-IDTBR vs annealed PM6:o-IDTBR, all absorption-corrected for 400 nm excitation. Grazing-incidence wide-angle X-ray scattering (GIWAXS) results for (f) o-IDTBR as-prepared, (g) annealed o-IDTBR; (h) PM6:oIDTBR as-prepared; (i) PM6:o-IDTBR annealed. (j) Resonant soft X-ray scattering (RSoXS) profiles of PM6:o-IDTBR blends at nonresonant (270 eV) and resonant (285.1 eV) photon energies.

The photoluminescence (PL) (Figure 1d and e) of the as-prepared o-IDTBR film reveals a peak at 750 nm which undergoes a redshift to 820 nm upon annealing, accompanied by a moderate decrease in intensity. The difference in PL intensity observed within these neat films serves as an indicator of different nonradiative processes taking place due to differences in aggregation and crystallinity between the thermally annealed and as-prepared samples. The latter is supported by our morphology data (see Figure 1 and SI figure S1) showing larger crystalline aggregates of o-IDTBR in annealed neat film. In the blends, however, a broad PL band between 760 and 870 nm is observed for both as-prepared and annealed films (with a small change in the intensity), resulting in PL quenching of 40% for the as-prepared and 50% for the annealed blend. The corresponding PL quantum yields (PLQY) are given in Table 1. Neat o-IDTBR exhibits one of the highest reported PLQY values (for the relevant materials) of 12.5% (as-prepared film and 10.6% for the annealed film); rigidly planar molecules tend to have greater fluorescence quantum yields due to the deactivation of nonradiative relaxation. Similarly, the blend films both have similar quantum yields, yielding poor exciton dissociation of only about 40–50% for the as-prepared and the annealed blends. Intriguingly, morphology data suggest the blends have reduced aggregation compared to the neat film; however, the annealed blend still exhibits some aggregation but with a similar relative purity, as obtained from RSoXS (Figure 1 and Figure S1). These results are consistent with PM6:o-IDTBR being a small offset system, lacking sufficient driving force to efficiently dissociate excitons at the interface.^26^

**Table 1:** Photoluminescence Quantum Yield (PLQY) of annealed and as-prepared neat o-IDTBR and blend PM6:o-IDTBR films^a^

**Sample**	**PLQY (%)**	**PL quenching (%)**
o-IDTBR (as-prepared)	12.5	-
o-IDTBR (annealed)	10.6	-
PM6:o-IDTBR (as-prepared)	5.1	40%
PM6:o-IDTBR (annealed)	5.2	50%

a PL quenching obtained from PL of blend relative to the neat films.

To identify which excited states under standard operating conditions at steady state contribute to the device performance, photoinduced absorption (PIA) spectroscopy was employed. Figure 2a illustrates the spectrum of a annealed PM6:o-IDTBR blend semitransparent device. The spectral analysis reveals three distinct features. First, we observe ground state bleach (GSB) between 500 to 650 nm, corresponding to both PM6 and o-IDTBR. Second, the electroabsorption (EA) of PM6 is within the wavelength range of 650 to 800 nm, aligning with previous findings.^25^ Lastly, a broad photoinduced absorption (PIA) band is observed, peaking in the 800 to 1000 nm region. Notably, no detectable signal is observed above 1000 nm. Since PIA is conducted under conditions corresponding to hundreds of ns to μs time-scale, the PIA peak is ascribed to long-lived species which can include free carriers and triplets but not excitons or CT states, which only exists at the 100–1000 ps time scale. The broad PIA spectrum hints at presence of two species: triplet excitons and free carriers. In order to discern and characterize these species effectively, the spectrum was taken at steady state conditions at 570 Hz frequency under nitrogen and ambient environments. Notably, the PIA region of the spectra manifests differences under varying frequency and environmental conditions.

**Figure 2:**
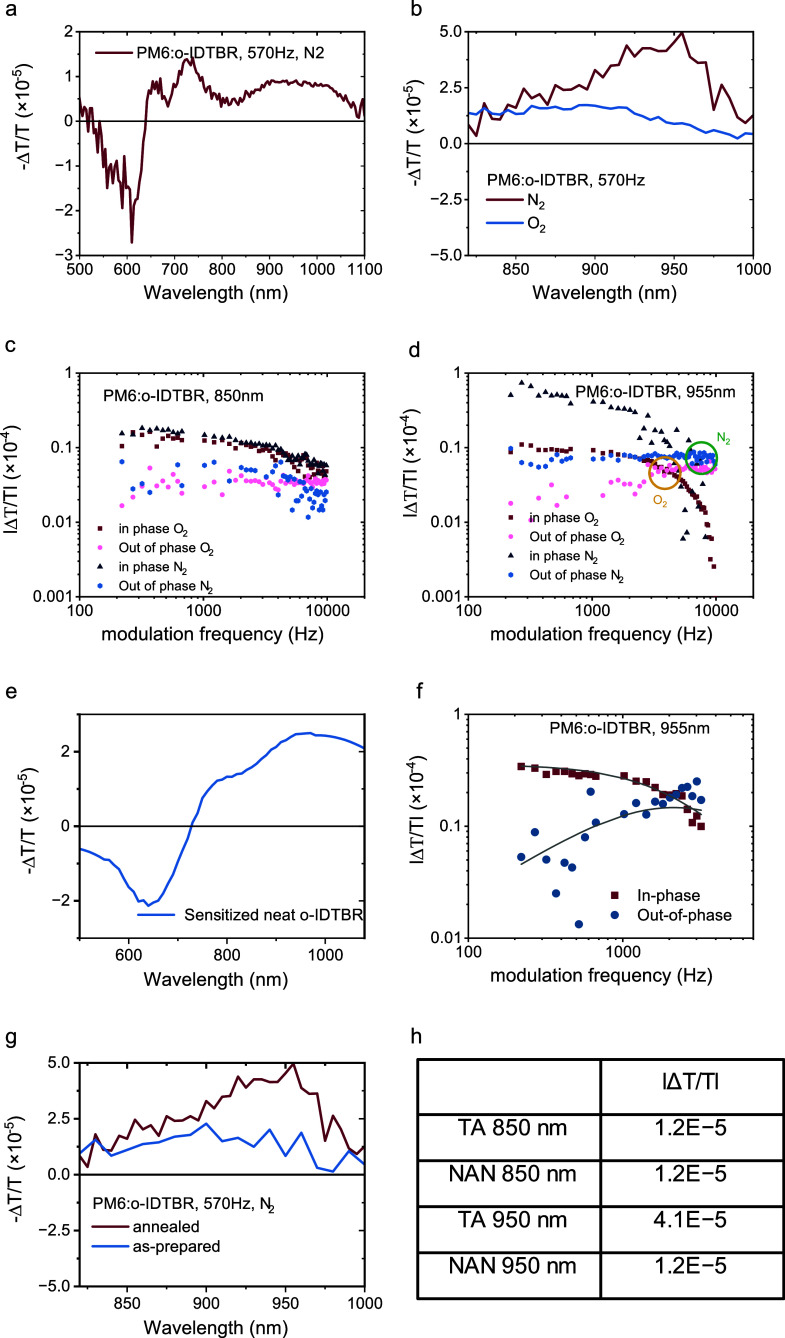
Photoinduced absorption (PIA) spectra of semitransparent PM6:o-IDTBR solar cells, (a) annealed, measured at 570 Hz under N_2_ atmosphere, (b) annealed sample under N_2_ and O_2_ atmospheres. Frequency modulation: in-phase and out-of-phase components of annealed PM6:o-IDTBR sample: (c) 850 nm peak and (d) 955 nm peak. (e) PIA spectrum of neat o-IDTBR sensitized with PtOEP, under N_2_ atmosphere. (f) Modulation frequency of sensitized neat o-IDTBR film, probed at 955 nm. (g) PIA spectra of annealed and as-prepared PM6:o-IDTBR solar cells at 570 Hz under N_2_ atmosphere. (h) Summary of amplitude of the two peaks in the annealed blend (TA) and the as-prepared (NAN) blend.

Turning to semitransparent devices and concentrating on the PIA peak only, under nitrogen, at 570 Hz (Figure 2b), the spectrum consists of a broad peak with a feature at 850 nm and one at 950 nm with the latter dominating in amplitude. Under oxygen (at 570 Hz), the feature at 950 nm has diminished, and the amplitude of the 850 nm feature remains constant (Figure 2b). Furthermore, the kinetics of each band under nitrogen and oxygen environments are probed (Figure 2c,d). In the absence of degradation, the decay dynamic for the 850 nm band is insensitive to oxygen, while the decay dynamic of the 955 nm species is accelerated under O_2_. Given that the presence of oxygen leads to a strong and, importantly, reversible reduction in the amplitude and lifetime of the 950 nm band, we assign this feature to the triplet excitons. This is because oxygen reacts with triplet-excited states, leading to a reduction in the triplet population according to the energy transfer reaction
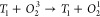


To substantiate the assignment of triplets, Figure 2e shows the spectrum of neat o-IDTBR sensitized with platinum octaethylporphyrin (PtOEP). Upon exciting PtOEP, PtOEP triplets are generated, subsequently undergoing energy transfer to yield the lower-energy o-IDTBR triplets. The spectrum (panel e) reveals a peak centered at 950 nm, aligning with the feature in the PM6:o-IDTBR spectrum present exclusively under the N_2_ condition and not under O_2_. The decay of the sensitized film, as illustrated in Figure 2f, reveals a measured lifetime of 10 μs assigned to the o-IDTBR triplet.

We assign the remaining PIA band at 850 nm, which is insensitive to oxygen, to free charges (CS). Figure 2g compares the spectra of the annealed blend with that of the as-prepared blend. Notably, there is a significant difference in the amplitude of triplets at 950 nm between the two samples (almost absent in the nonannealed), whereas the 850 nm peak, indicative of the free charge carrier density, remains comparatively consistent (panel h summarizes the amplitudes). The triplet absorption cross-section was determined using the GSB of the sensitized o-IDTBR film (see Supplementary Note 4) which was used to determine the triplet population in the PM6:o-IDTBR as 6 × 10^15^ cm^–3^ for the annealed and 1.5 × 10^15^ cm^–3^ for as-prepared blends. Thus, the triplet recombination rate, *R*, can be determined using the triplet population and its characteristic lifetime. In the annealed sample, the calculated recombination rate for the triplet channel is 6 × 10^20^ cm^–3^ s^–1^ in comparison to the total recombination rate of 4.35 × 10^21^ cm^–3^ s^–1^ derived using the free charge population and recombination rate of free charges.^26^

For a better comprehension of how T1 and its population could influence the voltage, we conducted temperature-dependent *V*_*OC*_ measurements (Figure 3a) and analyzed recombination rate equations. From extrapolation of *V*_*OC*_ to *T* → 0 K, *V*_0_ is obtained. The extrapolation of the *V*_*OC*_ from a given *T* to 0 K provides insight into the energy of the state primarily responsible for recombination at that temperature. It is important to recognize the inherent complexity of measurements in disordered systems, where energetic disorder can cause the entire data set to experience a downward shift, reducing *V*_0_ as well as influencing deviation from linearity at low temperatures. However, these effects are intrinsic to the system and form part of the recombination mechanism. The extrapolated *V*_*OC*_ at 300 to 0 K is now compared with the estimated *E*_*CT*_. Since in low offset systems determination of *E*_*CT*_ from EQE measurements is not possible (EQE_PV_ and EQE_EL_ spectra are dominated by the singlet state and not the CT state - see Supplementary Note 5 and Figure S4), we combined the optical gap energy with the energy barrier between the S1 and CT state obtained from temperature-dependent electroluminescence measurements (EL), in order to estimate the *E*_*CT*_ (see Supplementary Note 5 and Figure S4 for full detail). In both systems we observe that *V*_0_ is located approximately 0.2 eV below the estimated *E*_*CT*_, which could be lowered due to energetic disorder; therefore, we tentatively assign it to the CT state. This indicates that at room temperature, free charge recombination predominantly goes via the CT state. This is consistent with the ideality factor obtained from light-intensity-dependent Voc, with only small deviation from the ideal case of 1, which may be due to energetic disorder). The observation that *V*_0_ is not limited by triplets suggests efficient dissociation of triplet exciton to the triplet CT state, and so, the recombination to the ground state is still mediated by the CT state. In this scenario the *V*_*OC*_ is described as commonly done by *E*_*CT*_ (see Supplementary Note 6 for rate equation for the case of triplet vs CT limited recombination) and not by the triplet energy level which in case of dominant recombination through T1 would read as
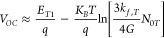
where *E*_*T*1_ is energy of the excitonic triplet state, *k*_*f,T*_ is the decay of the triplet state to the ground state (also referred to as phosphorescence), and *N*_0*T*_ is the density of the triplet state.

**Figure 3:**
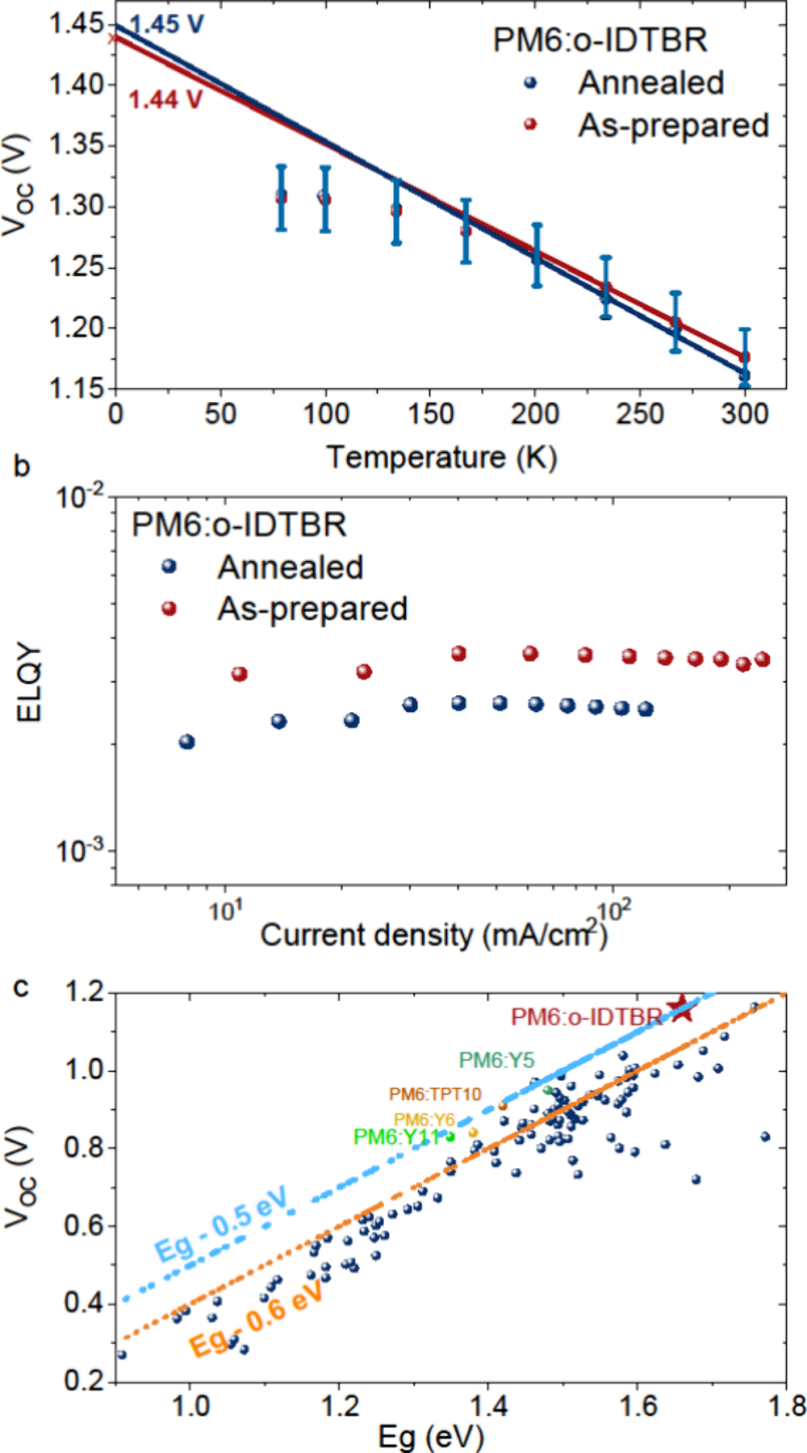
(a) Temperature-dependent open-circuit voltage (*V*_*OC*_–*T*) characteristics of as-prepared (red) and annealed (blue) PM6:o-IDTBR devices. Solid lines represent linear fits to the measured data. (b) Electroluminescence quantum efficiency (ELQY) as a function of applied current density for as-prepared (red) and annealed (blue) PM6:o-IDTBR devices. (c) Graphical representations of photovoltaic bandgap (*E_g_*) and open-circuit voltage (*V*_*OC*_) for a range of systems.^29−38^

We note that Vandewal and co-workers also investigated the potential role of triplet states in donor or acceptor materials in contributing to voltage losses.^23^ Their findings indicate that, in the systems studied, which included Zn- and Cu-phthalocyanine donors, the relative energy alignment between the T1 state and the CT state does not significantly impact the *V*_*OC*_; however the reason behind this was not examined or discussed.

When triplet state is the lowest energy state, it is expected to induce significant nonradiative voltage losses.^8^ However, these losses can be mitigated by suppressing triplet state decay, thereby preventing *V*_0_ from being constrained by *E*_*T*_. This can be achieved if the T1 state rapidly dissociates back to ^3^CT state and eventually generating free carriers. It is important to highlight that, in this scenario, the lifetimes of T1 must exceed that of the ^1^CT, while the energy levels are not too far apart. Under these conditions, T1 population and energy level become inconsequential for *V*_*OC*_, effectively preventing the additional nonradiative voltage losses.^8^ When the decay rate of the CT state exceeds that of phosphorescence (*k*_*f,T*_), the T1 state mostly assumes the role of a reservoir for the CT state, establishing an equilibrium with both CT and the CS state. This equilibrium effectively precludes the emergence of an extra loss pathway through triplets (depending on the ratio of *k*_*k*_*f,T*__/*k*_*f*_). Indeed, to a large extent this may be the case in the annealed PM6:o-IDTBR device, which exhibits exceptionally low *V*_*nr*_ losses. Both experimental measurements and calculations (see Supplementary Note 7 and Figure S6) show these losses to be just 160 meV, getting closer to silicon solar cells, which stand at 120 meV.^27^ In the case of the annealed PM6:o-IDTBR, the recombination rate of the triplet channel accounts for only 10% of the total recombination. This indicates that while triplets do participate in the recombination process, they do not play a significant role in this system. We note that, under the 1 sun condition, contribution from triplet–triplet annihilation is ruled out given the constant external quantum electroluminescence (ELQY) with increasing current (Figure 3b). In addition, the absence of any long-lived delayed fluorescence from our previously reported time-resolved PL data^26^ also rules out thermally activated delayed fluorescence in this system. Furthermore, the comparison between the annealed and the as-prepared blends further substantiates the insignificant role of triplets in this system. The EQE_EL_ data measure identical nonradiative voltage loss across both systems (see Figure 3b), despite differences in the T1 population, as obtained from PIA (see Figure 2). In Figure 3c, we present graphical representations of *V*_*nr*_ and *V*_*OC*_ for a range of systems described in the literature, alongside our study on PM6:o-IDTBR. While PM6:o-IDTBR shows less loss on these plots, reminiscent of *E*_*CT*_-0.5 rather than the previously observed *E*_*CT*_-0.6, the data still hint at a possible connection between the characteristics of the CT state in describing the *V*_*OC*_ even when considering lower loss scenarios.

In order to mitigate the decay of triplet excitons and the accompanying voltage loss, a viable strategy involves reducing the energy difference between the CT and the triplet states, thus facilitating the dissociation rate of triplet excitons to the CT state. The process of aggregation, particularly within the donor material, has been suggested as an effective means to achieve this objective. Chen et al. have previously demonstrated that aggregation induces interchain charge delocalization, resulting in a lowering of the CT energy.^45^ This reduction in the CT energy proves instrumental, as it enhances the efficiency of triplet exciton dissociation back to the CT state. Notably, when the CT energy is reduced to a point where it is smaller than T1, a barrier arises between these two states. This barrier mitigates the possibility of back electron transfer from the CT state and potentially impedes the formation of triplets. A schematic representation of triplet exciton dissociation and back electron transfer processes in solar cells is shown in Figure 4. Thermal annealing is frequently employed to promote aggregation within a system. In the case of PM6:o-IDTBR blends, although some variations in PL and domain size are observed between the as-prepared and thermally annealed films, their impact on aggregation in the blend is minimal. This is evidenced by the nearly identical absorption spectra of the two blend films, which is consistent with the similar *E*_*CT*_ values estimated for both blends. This observation aligns with findings by Chen, which suggest that acceptor aggregation has a less pronounced effect on the CT energy than polymer aggregation. As such in this system, the reduction in triplet generation in the as-prepared blend is not driven by changes in the relative energies of the CT and triplet states but rather by other mechanisms perhaps such as a change in the triple energy level, which require further investigation.

**Figure 4:**
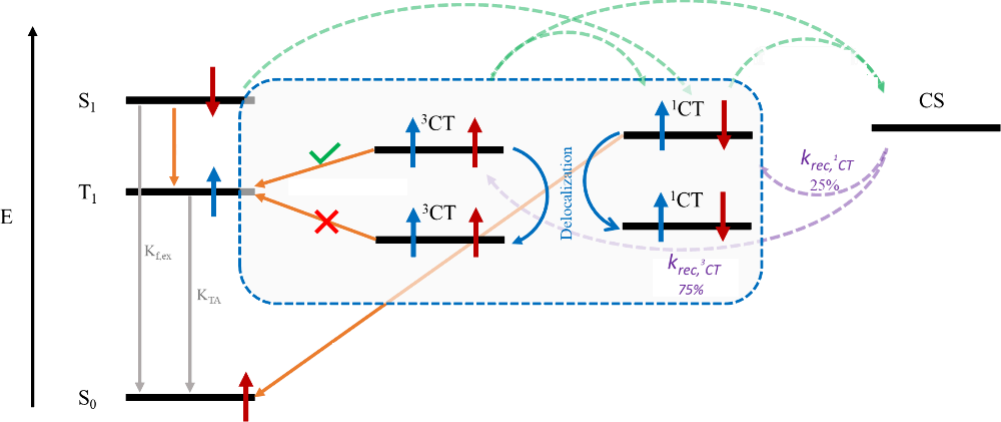
Schematic diagram illustrating the impact of aggregation-induced interchain charge delocalization, on triplet exciton dissociation and CT state energy. When aggregation is strong enough, it pushes the CT state to be the lowest state and thereby creates a barrier for back electron transfer.

Switching from PM6 to PTQ10, a more aggregated polymer,^46^ can provide insight into this direction. However, the deeper HOMO level of PTQ10 compared to both PM6 and o-IDTBR complicates the determination of the S1–CT offset and, consequently, *E*_*CT*_. Furthermore, the absence of a favorable energy offset precludes exciton quenching. On the other hand, ITIC-4F has a deeper HOMO level and is also known to exhibit long-lived triplets in the 900–1200 nm region.^47^ Our PIA measurements on blends of PM6:ITIC-4F and PTQ10:ITIC-4F (see Supporting Information Figure S7) confirm the presence of ITIC-4F triplets in both blends but more prominently in the PM6 blend. In the PTQ10 blend, we attribute the lower triplet population to the more aggregated nature of PTQ10, perhaps affecting the CT energy relative to the T1 and thus reducing the rate of triplet formation, *k*_*BET*_. However, switching from o-IDTBR to ITIC-4F blends, it should be pointed out that the deeper HOMO energy level results in the observation of the CT state (in EL measurements as previously observed in the large offset systems) and thus higher nonradiative voltage losses.

In conclusion, our study offers insights into the energy-gap-law dependence, even in the presence of a triplet state. We estimate the total recombination rate of the triplet channel and the CT channel to elucidate the characteristics of open-circuit voltage and the mechanisms behind nonradiative voltage losses with respect to triplet excitons. Our collective empirical findings on the PM6:o-IDTBR system, from PIA spectroscopy, photovoltaic characterization, and detailed energy loss analysis, shed light on the presence of long-lived triplet excitons; yet, the triplet recombination channel contributing only about 10% of the total recombination of the free charges over the CT state. The lower energetic position of *E*_*T*_ relative to *E*_*CT*_ plays a secondary role in determining Δ*V_nr_* and the overall *V*_*OC*_ losses as the long triplet lifetime allows the triplet excitons to dissociate into the CT state, thereby minimizing their impact on nonradiative voltage losses. Ultimately, we provide two design rules to minimize losses from triplets: long triplet lifetimes and engineering the energy offset between CT and T1 states perhaps with enhanced delocalization of the CT state to reduce the CT energy. The latter will result in 3 improvements: decelerate BET from ^3^CT to T1, facilitate T1 dissociation to ^3^CT and subsequently to the CS state, as well as assist singlet exciton dissociation to ^1^CT.

## Supporting Information Available

Details on materials and device preparation and measurement techniques, morphology data, triplet density calculation and demonstrating recombination pathways, calculation of nonradiative voltage, PIA data on different systems (PDF)
