# Supplementary material for: When the Triplet State Doesn’t Matter: Insights into Its Impact on VOC
Source: ACS Energy Lett. 2025 Apr 23;10(5):2419–27. doi: 10.1021/acsenergylett.5c00384 (PMC12070747; doi:10.1021/acsenergylett.5c00384)
Supplement: Supplementary file 1 — nz5c00384_si_001.pdf [file nz5c00384_si_001.pdf]

SUPPORTING INFORMATION to the manuscript:

When the Triplet State Doesn't Matter: Insights into Its Impact on V_{OC}

Mohammad Saeed Shadabroo[1], Nurlan Tokmoldin[2], Atul Shukla[1], Acacia Patterson[3], Tanner M. Melody[3], Obaid Alqahtani[3,4], Brian A. Collins[3], Dieter Neher[2], Safa Shoaee[1,2*]

[1] Institute of Physics and Astronomy, University of Potsdam, Karl-Liebknecht-Str. 24-25, 14476 Potsdam-Golm, Germany

[2] Heterostructure semiconductor physics, Paul Drude Institute for Solid State Electronics, Hausvogteiplatz 5-7, 10117 Berlin, Germany

[3] Department of Physics and Astronomy, Washington State University, 100 Dairy Road, Pullman, WA 99164, USA.

[4] Department of Physics, Prince Sattam bin Abdulaziz University, Alkharj, 11942, KSA

Supplementary Note 1 | Details on materials and device preparation and measurement techniques

The photoactive blends in this study utilized the following organic materials:

Poly[(2,6-(4,8-bis(5-(2-ethylhexyl-3-fluoro)thiophen-2-yl)-benzo[1,2-b:4,5- b']dithiophene))-alt-(5,5-(1',3'-di-2-thienyl-5',7'-bis(2-ethylhexyl)benzo[1',2'-c:4',5'- c']dithiophene-4,8-dione)] (**PM6**) served as the donor.

(5Z,5'Z)-5,5'-((7,7'-(4,4,9,9-tetraoctyl-4,9-dihydro-s-indaceno[1,2-b:5,6-b']dithiophene-2,7-diyl)bis(benzo[c][1,2,5]thiadiazole-7,4- diyl))bis(methanylylidene))bis(3-ethyl-2-thioxothiazolidin-4-one) (**o-IDTBR**) was employed as the acceptor.

All devices in the investigation were constructed on indium-tin oxide (**ITO**)-coated glass substrates. The photovoltaic cells followed a conventional architecture of ITO/ hole-transport layer (HTL)/ blend layer/ electron-transport layer (ETL)/ Ag. The HTL comprised a 30 nm layer of poly(3,4-ethylenedioxythiophene) polystyrene sulfonate (**PEDOT:PSS**), spin-coated in air, and annealed at 150°C for 15 minutes. The ETL consisted of a 15 nm layer of N,N'-bis{3-[3-(dimethylamino)propylamino]propyl}perylene-3,4,9,10-tetracarboxylic diimide (**PDINN**), spin-

coated in nitrogen (N2) on top of the photoactive blend layer. The top Ag electrode, with a thickness of 100 nm, was thermally evaporated under a base pressure of 1×10-7 mbar.

The blends of D:A were dissolved in chloroform at a donor-to-acceptor weight ratio of 1:1.2, with a total concentration of 16 mg/mL, resulting in an approximately 120 nm film. Following spin-coating, the annealed film underwent thermal annealing at 110°C for 10 minutes in N2, whilst the non-annealed was subject to no treatment.

Current Density-Voltage (J-V) Measurements: J-V measurements were carried out in an N2 atmosphere using a Newport Oriel Sol2A solar simulator equipped with temperature control and a Keithley 2400 SourceMeter. The illumination intensity was adjusted to AM1.5G conditions employing a calibrated Si solar cell.

Ultraviolet-Visible Absorption (UV-Vis) Spectroscopy: Optical absorption measurements were conducted using a Cary 5000 UV-Vis-NIR spectrophotometer, which was equipped with an integrating sphere to account for reflectance.

External Photovoltaic Quantum Efficiency (EQE-PV): The Incident Photon-to-Electron Conversion Efficiency (IPCE) spectrum of the studied solar cells was characterized using an SR 830 DSP lock-in amplifier. This measured the photoinduced signal produced in the studied cell by monochromated illumination from a halogen lamp. The measurements were normalized to the IPCE spectra of standard Si (300 to 1100 nm) and Ge (1000 to 1400 nm) photodiodes.

Photoluminescence (PL) and Electroluminescence (EL): PL measurements of the studied blends in complete devices under various applied biases were performed using an Andor Shamrock SR303i-B spectrograph equipped with a DU420A-BR-DD Si detector (for a central wavelength of 800 nm) and a DU491A-1.7 InGaAs detector (for central wavelengths of 1100 and 1400 nm). The applied bias was controlled using a Keithley 2400 SourceMeter. PL Quantum Yield (PLQY) measurements on PS:NFA films, with the same ratio as the studied polymer blends, were conducted inside an integrating sphere (Hamamatsu Photonics K.K. A10094) under steady-state illumination from a 520 nm continuous wave laser (InsaneWare). EL Quantum Yield (ELQY) measurements were performed by applying forward bias to the studied solar cells via a Keithley 2400 SourceMeter and detecting outgoing emission using a Si photodiode.

Quasi-Steady-State Photoinduced Absorption (PIA): In quasi-steady-state PIA measurements, the pump beam emitted from a continuous-wave laser diode was modulated by an optical chopper with

a frequency tuneable in the range of 200 Hz to 10 kHz. This modulated beam was focused on the PM6:o-IDTBR solar cells with a spot diameter of approximately 3 mm. The excitation wavelength was set to 405 nm (3.06 eV), and the excitation fluence could be adjusted using a programmable attenuator. Simultaneously, a tungsten halogen lamp served as a source for the probe beam. The lamp's white light was directed to a monochromator to extract monochromatic probe light at different wavelengths. The monochromatic probe beam, exiting the monochromator, was directed to spatially overlap with the modulated pump beam on the sample's surface. The change in the transmitted probe beam induced by the modulated photoexcitation was detected using a Si or InGaAs photodiode, depending on the probe wavelength, and a lock-in amplifier referenced at the modulation frequency of the pump beam. The PIA signal was corrected by subtracting the photoluminescence background. Our system can achieve a sensitivity as low as 5×10^{-7} for PIA measurements.

Morphology: Grazing-incidence wide-angle X-ray scattering (GIWAXS) measurements were conducted on samples of neat and blend films that were cast on silicon substrates. The GIWAXS measurements were acquired with the Xenocs XEUSS 3.0 that utilized a focused Cu source at an incident angle of 0.25 degrees (above the critical angle) with 30 minute exposures. 1D GIWAXS profiles were processed via NIKA software.

Resonant soft X-ray spectroscopy (RSoXS) was performed at NSLS-ii SST1_7-ID-1 and processed via custom Igor Pro software.

Atomic force microscopy (AFM): was used to probe the surface roughness of the studied samples via a Bruker’s Dimension Icon microscope, using a nitride lever with a SCANASYST-AIR silicon tip (f_0= 70 kHz and k=0.4 N/m). The Gwyddion software was used to process the AFM scans and obtain the root-mean square (RMS) roughness values. The AFM samples were cast on Si substrates.

Transmission electron microscopy (TEM): was conducted in bright-field mode with a 200 kV source and CCD detector (FEI Eagle 4k), FEI Technai G2 20 Twin (Thermo Fisher) microscope. Thin films of the examined samples were floated off in deionized water then mounted onto TEM grids.

Supplementary Note 2 | Current Voltage parameters

Table S1. JV parameters for annealed and as-prepared PM6:o-IDTBR

Device	V_{OC} (V)	J_{SC} (mA/cm^2)	FF (%)	PCE (%)
PM6:o-IDTBR annealed	1.16	8.64	52.9	5.5
PM6:o-IDTBR as-prepared	1.17	6.88	50.5	4.2
PM6:o-ITIC-4F	0.87	18.02	57.3	9.7
PTQ10:ITIC-4F	0.93	19.76	66.2	13.5

Supplementary Note 3 | Morphology

All morphology data consistently point towards much larger aggregates of o-IDTBR in annealed neat film. In blend films the morphology is more similar, however the thermally annealed blend still exhibits somewhat more aggregation. RSoXS analysis at the resonant energy of 285.1 eV shows similar mesoscale domains with characteristic length of 40-50 nm.

By integrating the Lorentz-corrected profiles, we obtain total scattering intensity which is proportional to the square difference in concentration between domain type 1 and 2 (say donor concentration difference between each the donor-rich and acceptor-rich domains). Thus the concentration difference between domains in a sample is proportional to the square root of the integral of the RSoXS profiles. By taking the ratio of these results between the two samples we can arrive at a relative purity between the two. From this we calculate that they are both very close together in purity with the as-prepared sample at 97% the purity of the annealed sample (only 3% less pure).

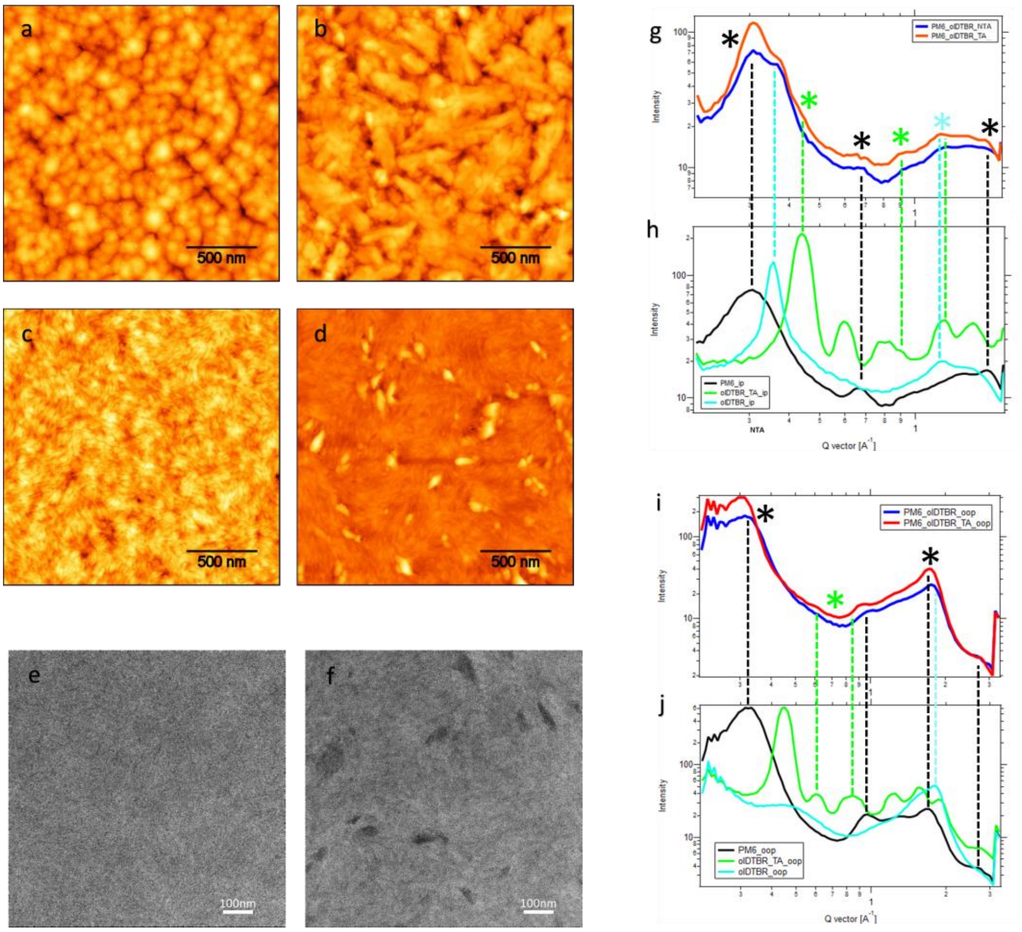

Figure S1. Atomic force microscopy (AFM) images: (a) as-prepared neat o-IDTBR, (b) thermally annealed neat o-IDTBR, (c) as prepared PM6:o-IDTBR blend, and (d) thermally annealed PM6:o-IDTBR blend. Transmission electron microscopy (TEM) scans of (e) as prepared PM6:o-IDTBR blend and (f) thermally annealed PM6:o-IDTBR blend. GIWAXS profiles extracted from GIWAXS 2D scans (g) in-plane profiles of PM6:oIDTBR blends; (h) in-plane profiles of pure components; (i) out-of-plane profiles of PM6:oIDTBR blends; (j) out-of-plane profiles of pure components.

Supplementary Note 4 | Triplet density calculations

In order to obtain the steady state triplet density in the blend, we first calculate the triplet population in the sensitized o-IDTBR film and thereby obtain the absorption cross section of o-IDTBR triplets.

$$OD = \frac{\text{Number of molecules}}{V} \times \sigma \times d$$

$$\frac{N}{V}\left[\frac{molecules}{cm^3}\right] = c_M\left[\frac{mol}{cm^3}\right] \times N_A\left[\frac{molecules}{mol}\right]$$

N_A Avogadro's constant

c_M the molar concentration (1329 $\frac{mol}{liter}$)

M_n molecular weight (1326 g/mol)

Number of molecules (in OIDTBR (d=150nm)) : $\frac{N}{V}\left[\frac{molecules}{cm^3}\right] = 3.6 \times 10^{20}\left[\frac{molecules}{cm^3}\right]$

$$\Delta OD_{GSB} = \frac{\text{Nexcited}}{V} \times \sigma \times d$$

$$\frac{\Delta \text{OD}_{GSB}}{\text{OD}} = \frac{\text{Nexcited}}{\text{Number of molecules}}$$

Nexcited: the number of molecules which are no more in the ground state due to the number of triplet population in the sensitized film

$$\frac{2.13 \times 10^{-5}}{0.5 \times ln10} = \frac{\text{Nexcited}}{3.6 \times 10^{20}}$$

In the sensitized neat o-IDTBR film

$$Nexcited = n_{triplet}$$

$$\frac{\Delta T}{T} = (ln10) \times n \times d \times \sigma$$

$$\sigma_{o-IDTBR} = 2.2 \times 10^{-16}\ cm^2$$

In the PM6:O-IDTBR blend,

$$n_{triplet} = 6 \times 10^{15}\ cm^{-3}$$

Supplementary Note 5 | Estimating the E_{CT} from temperature dependent electroluminescence measurements and optical bandgap

PM6:o-IDTBR exhibits small offset. The absorption and emission from the S1 completely dominate the photovoltaic and electroluminescence external quantum efficiency – with an exciton reformation efficiency of about 3%, and that no information on the energetics of the CT state manifold can be gained from these spectra (Figure S4).

In a T-EL measurement, as with the injection current held constant, the number of reformed S_1 states is essentially determined by the competition between the CT decay rate and the net S_1 repopulation rate from the CT state. While the CT decay rate is described by a constant k_f and independent of temperature, the S_1 reformation rate from CT can be represented by the exponential expression:

$$k_{ref} = k_{ref}^{*} \times \exp(-\frac{\Delta E_{s_1-CT}}{K_B T})$$

where k_{ref}^{*} corresponds to the net CT-S_1 reformation rate k_{ref} at infinite temperature, ΔE_{s_1-CT} is the energy offset between the S1 and CT states, K_B Boltzmann constant and T temperature. Since the EL spectra in the studied systems are almost entirely represented by exciton emission, the EL peak intensity values are proportional to the number of reformed excitons from the CT state, during the charge injection process:

$$p \sim e^{-\frac{\Delta E_{s_1-Ct}}{KT}}$$

$$I_{EL} = I_{0_{EL}} \times e^{-\frac{\Delta E_{s_1-CT}}{KT}}$$

$$ln(I_{EL}) = ln\left(I_{0_{EL}}\right) - \frac{\Delta E_{s_1-CT}}{KT}$$

Therefore, the slope of $ln(I_{EL})$ and reciprocal of T yields a $-\frac{\Delta E_{s_1-CT}}{K}$ value, providing valuable insight into the energy difference between the S1 and CT. To ascertain the CT energy, we need to obtain the S1 energy. This is accomplished through the derivation of the optical band gap from the absorption and photoluminescence of the films. The difference between these values results in an estimate of E_{CT}.

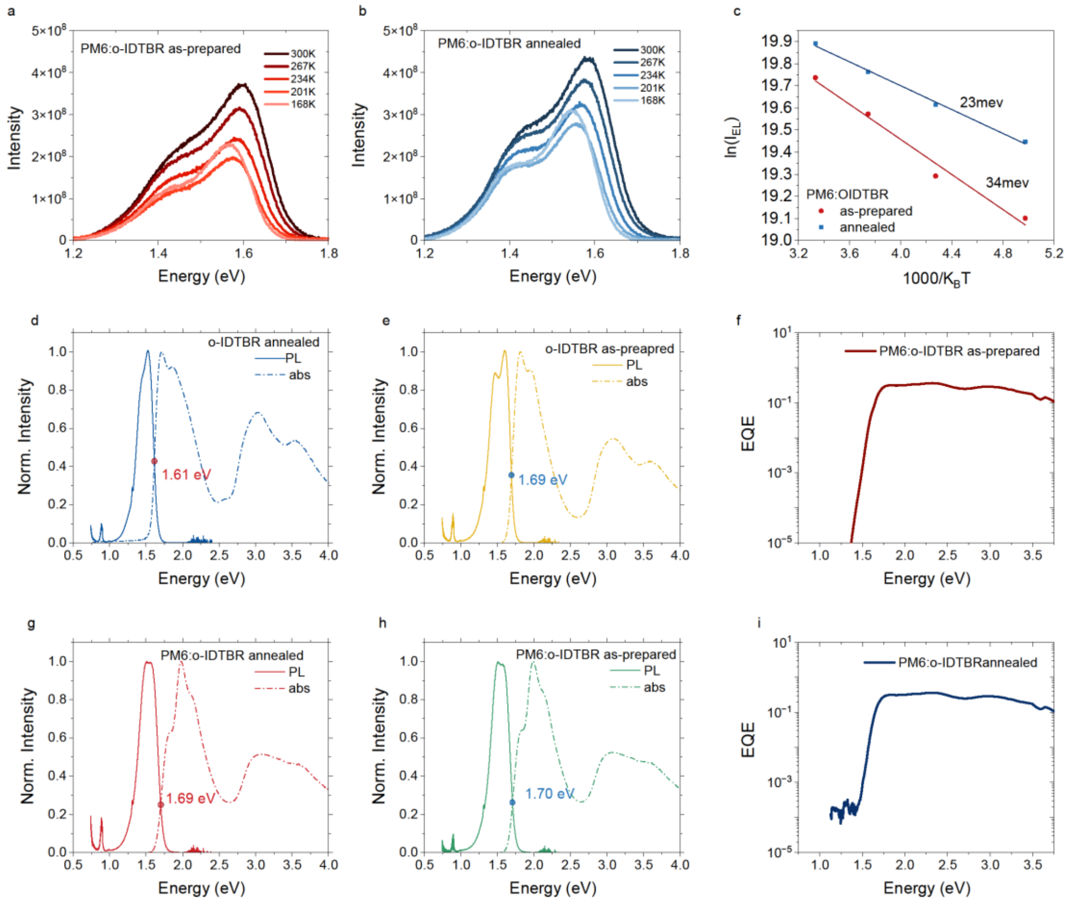

Figure S4 (a, b) Temperature dependent EL spectra measured in Cryostat for as-prepared PM6:o-IDTBR and annealed PM6:o-IDTBR. (c) Temperature-dependent EL peak intensity of the devices. Normalized absorption and PL (d) annealed neat o-IDTBR, (e) as-prepared neat o-IDTBR, (g) annealed PM6:o-IDTBR and (h) as-prepared PM6:o-IDTBR. EQE spectra for (f) as-prepared PM6:o-IDTBR and (i) annealed PM6:o-IDTBR.

Table S2 Determination of CT energy, E_{CT} as explained above. E_{opt} is the optical bandgap obtained from intersection of absorption and emission, ΔE_{s_1-CT} is the energy gap between S1 and the CT state obtained from temperature dependent EL, and V_0 is the voltage at 0 kelvin extrapolated from temperature dependent open circuit voltage profile.

	E_{opt} eV	ΔE_{s_1-CT} eV	E_{CT} eV	V_0 eV
o-IDTBR TA	1.6	-	-	-
o-IDTBR NTA	1.7	-	-	-
Blend TA	1.7	0.023	1.68	1.45
Blend NTA	1.7	0.034	1.67	1.44

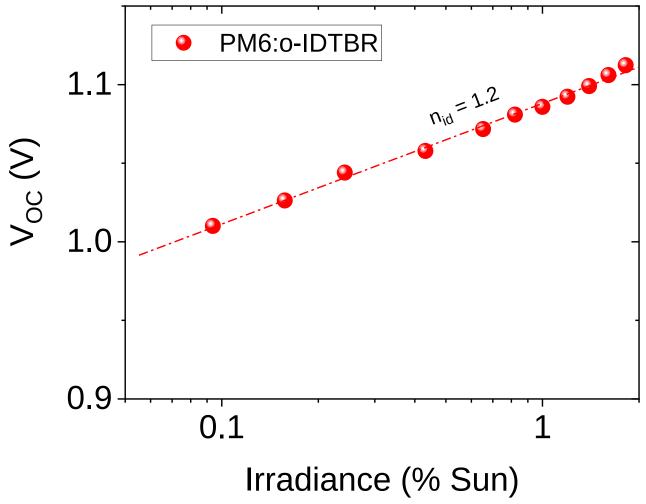

Figure S5 Voc as a function of light intensity. The slope gives an ideality factor of 1.2 for annealed PM6:o-IDTBR.

Supplementary Note 6 | Calculation demonstrating recombination pathway to the ground state

When triplet (T) formation is in equilibrium with its loss (decay and reformation of the CT state)

$$k_{f,T}N_T + k_{d,T}N_T = k_{BET}N_{CT3}$$

$$N_T = \frac{k_{BET}N_{CT3}}{k_{d,T} + k_{f,T}}$$

At V_{OC} all photogenerated carriers have to recombine $G = R$

$$R = k_{f,T}N_T + k_{f,CT1}N_{CT1} = G$$

$$N_{CT1} = \frac{1}{4}N_{CT} \text{ and } N_{CT3} = \frac{3}{4}N_{CT}$$

$$R = \frac{3}{4}k_{f,T}\frac{k_{BET}N_{CT}}{k_{d,T} + k_{f,T}} + \frac{1}{4}k_{f,CT}N_{CT} = G$$

$$R = \left[\frac{3}{4}k_{f,T}k_{BET} + \frac{1}{4}k_{f,CT}\left(k_{d,T} + k_{f,T}\right)\right]\frac{N_{CT}}{k_{d,T} + k_{f,T}} = G$$

If T1 and the CT state are coupled via a Boltzmann statistic, with the energy difference of $\Delta E_{CT-T} = E_{CT3} - E_{T1}$

$$k_{d,T} = k_{BET}\frac{N_{0CT}}{N_{0T}}exp - \frac{\Delta E_{CT-T}}{K_B T}$$

Density of CT states

$$N_{CT} = N_{0CT}\ exp\ \frac{qV_{OC} - E_{CT}}{K_B T}$$

If $k_{d,T} \geq k_{f,T}$

$$G = \left[\frac{3}{4}k_{f,T}\frac{N_{0T}}{N_{0CT}}exp\frac{\Delta E_{CT-T}}{K_B T} + \frac{1}{4}k_{f,CT}\right]N_{0CT}\ exp\ \frac{qV_{OC} - E_{CT}}{K_B T}$$

$$V_{OC} \approx \frac{E_{CT}}{q} - \frac{K_B T}{q}\ ln\left[\frac{k_{f,CT} + 3k_{f,T}\frac{N_{0T}}{N_{0CT}} exp\frac{\Delta E_{CT-T}}{K_B T}}{4G} N_{0CT}\right]$$

$$= \frac{E_{CT}}{q} - \frac{K_B T}{q}\ ln\left[\frac{k_{f,CT}N_{0CT} + 3k_{f,T}N_{0T} exp\frac{\Delta E_{CT-T}}{K_B T}}{4G}\right]$$

If dissociation of T is much faster than decay of the CT state: $k_{f,T}N_{0T}exp\frac{\Delta E_{CT-T}}{K_B T} \gg k_{f,CT}N_{0CT}$,$V_{OC}$ then becomes limited by the energy of the triplet state, E_T.

$$V_{OC} \approx \frac{E_T}{q} - \frac{K_B T}{q}\ ln\left[\frac{3k_{f,T}}{4G} N_{0T}\right]$$

This is exactly the case of all recombination running over the triplet state.

To show this we realize that the ratio of the triplet to CT recombination rate is

$$\frac{R_T}{R_{CT}} = \frac{k_{f,T}N_T}{k_{f,CT}N_{CT}} = \frac{k_{f,T}N_{0,T}}{k_{f,CT}N_{0,CT}}\ exp\frac{\Delta E_{CT-T}}{K_B T}$$

Then,

$$k_{f,CT}N_{0,CT} = \frac{R_{CT}}{R_T} k_{f,T}N_{0,T}\ exp\frac{\Delta E_{CT-T}}{K_B T}$$

$$qV_{OC} = \frac{E_{CT}}{q} - \frac{K_B T}{q}\ ln\left[\frac{k_{f,CT}N_{0CT} + 3k_{f,T}N_{0T} exp\frac{\Delta E_{CT-T}}{K_B T}}{4G}\right]$$

Which leads to

$$V_{OC} = \frac{E_{CT}}{q} - \frac{K_B T}{q}\ ln\left[\frac{\frac{R_{CT}}{R_T} k_{f,T}N_{0,T}\ exp\frac{\Delta E_{CT-T}}{K_B T} + 3k_{f,T}N_{0T} exp\frac{\Delta E_{CT-T}}{K_B T}}{4G}\right]$$

$$= \frac{E_T}{q} - \frac{K_B T}{q}\ ln\left[\frac{\left(\frac{R_{CT}}{R_T} + 3\right) k_{f,T}}{4G} N_{0T}\right]$$

When $\frac{R_{CT}}{R_T} < 1$

$$V_{OC} \approx \frac{E_T}{q} - \frac{K_B T}{q}\ ln\left[\frac{3k_{f,T}}{4G} N_{0T}\right]$$

Which is the above equation.

In the other limit for $\frac{R_T}{R_{CT}} < 1$

$$k_{f,T} N_{0,T}\ exp\frac{\Delta E_{CT-T}}{K_B T} = k_{f,CT} N_{0,CT}\frac{R_T}{R_{CT}}$$

which, when you put it into above equation leads to

$$qV_{OC} = \frac{E_{CT}}{q} - \frac{K_B T}{q}\ ln\left[\frac{k_{f,CT} N_{0CT} + k_{f,CT} N_{0,CT}\frac{R_T}{R_{CT}}}{4G}\right]$$

$$qV_{OC} = \frac{E_{CT}}{q} - \frac{K_B T}{q}\ ln\left[\frac{k_{f,CT} N_{0CT}}{4G}\right]$$

Where all recombination is dominated by the CT properties.

Supplementary Note 7 | Calculation and measurement of non-radiative voltage loss

$$\Delta V_{non-rad} = V_{oc,rad} - V_{oc}$$

$$V_{oc,rad} = \frac{k_B T}{q} \ln\left(\frac{J_{Ph}}{J_0}\right)$$

$$= \frac{k_B T}{q} \ln(\frac{J_{Ph}}{q \int EQEpv \, \phi_{BB} dE})$$

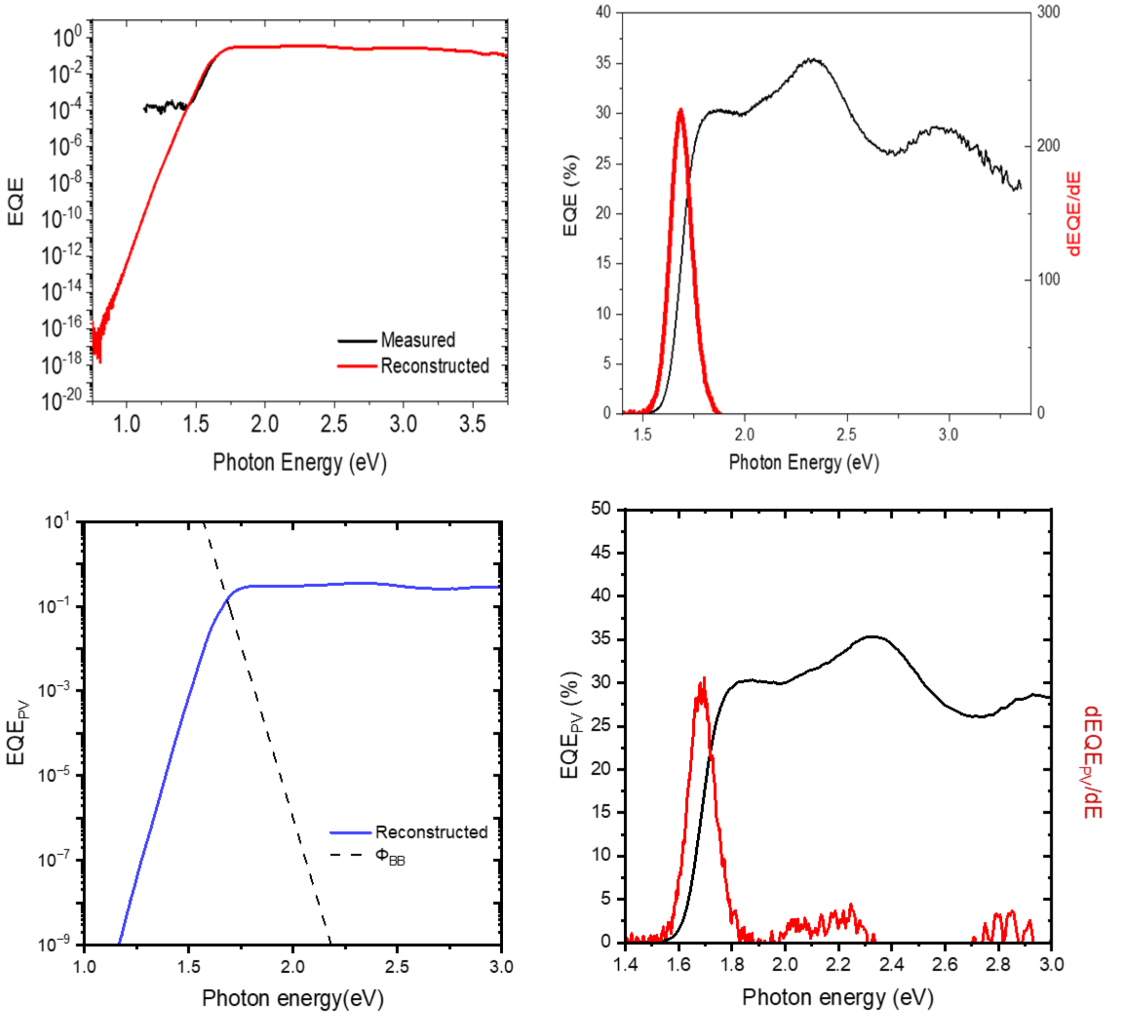

Figure S6 Top panel annealed PM6:o-IDTBR, bottom panel as-prepared PM6:o-IDTBR: photovoltaic external quantum efficiency measured and calculated (left), its derivative giving the photovoltaic bandgap of 1.68 eV (right)

Table S3 measured and calculated non-radiative voltage loss.

PM6: o-IDTBR	$J_{0,rad}$ (A/m^2)	E_g(eV)	$V_{OC,rad}$ (V)	$V_{OC,meas.}$(V)	$\Delta V_{nr.\text{meas}}$ (eV)	$\Delta V_{nr.\text{calc}}$ (eV)
TA	6.95×10^{-21}	1.68	1.30	1.15	0.16	0.169
As-prepared	6.93×10^{-21}	1,68	1,30	1,15	0,16	0.169

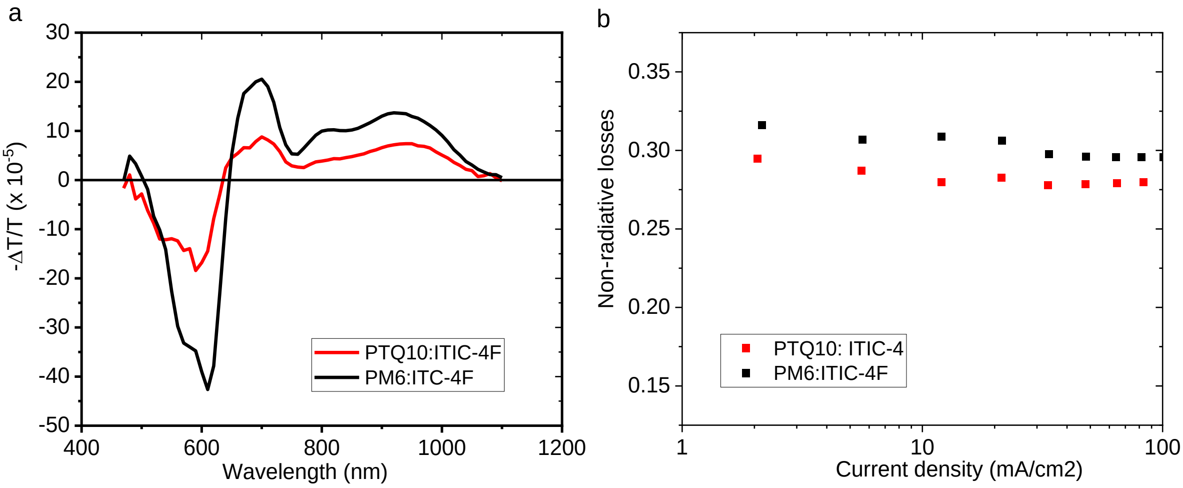

Figure S7 PIA spectra of PTQ10:ITIC-4F and PM6:ITIC-4F (a) and non-radiative voltage loss (b).
